# Epigenetic aberrations and cancer

**DOI:** 10.1186/1476-4598-5-60

**Published:** 2006-11-08

**Authors:** Miryam Ducasse, Mark A Brown

**Affiliations:** 1 Institute for Biomedical Research Georg-Speyer-Haus, 60596 Frankfurt, Germany; 2 Section of Molecular Genetics and Microbiology and Institute for Cellular and Molecular Biology, The University of Texas at Austin, 1 University Station A5000, Austin TX 78712, USA

## Abstract

The correlation between epigenetic aberrations and disease underscores the importance of epigenetic mechanisms. Here, we review recent findings regarding chromatin modifications and their relevance to cancer.

## Introduction

The development of tissues and organisms depends upon the acquisition of distinct programs for gene expression among individual cell types [1]. These programs are maintained in a heritable state by epigenetic mechanisms that impart cellular memory [2]. In this way, the global synchronization of patterns in gene expression broadly dictates developmental consequences [3]. At the core of such gene regulation are mechanistic pathways that affect the packaging of DNA into chromatin, thereby establishing the degree of DNA accessibility to transcriptional complexes [3-6]. These pathways include DNA methylation, chromatin remodeling, histone replacement, and alterations to histone tails [4,7,8]. Aberrations in these epigenetic mechanisms are known to be associated with the biology of cancerous lesions and their clinical outcome [1,9,10].

## Chromatin

From regulated gene expression to mitosis, chromatin acts as a structurally flexible repository of the genome [11]. In this manifestation, an entire chromosome is sequentially compacted through a series of highly ordered packaging while distinct regions of DNA are selectively made accessible to transcriptional complexes. Thus, chromatin maintains a dynamic architecture that allows approximately 2 m of DNA to be parceled in the nucleus while retaining a remarkable degree of functionality [12].

At its foundation, chromatin is grounded in a succession of nucleosomes, the basic structural unit [13], consisting of 146 base pairs of DNA, wrapped 1.7 times around an octamer of core histones and separated by a linker region of approximately 50 base pairs. The primary histones involved in the assembly of a nucleosome are histones H2A, H2B, H3 and H4. These histones form hetero-dimers such that each is represented twice in the nucleosome core unit [14].

The structure of each histone is highly conserved, including a folded core and an unstructured tail [15]. The histone core is a globular domain, forming a helix-turn-helix motif, which facilitates dimerization. Conversely, histone tails do not adopt defined conformations in crystal structures, except when bound to their recognition proteins [15]. These tail domains contain a number of conserved amino acid residues including lysine, arginine, and serine [16]. Histone tails, which sustain a basic charge, can interact with the poly-anionic backbone of the core DNA, marginally contributing to nucleosome stability [17]. Therefore, regulation of chromatin structure and transcription is often mediated through post-translational modifications that alter specific residues along these tails. These modifications can affect the accessibility of nuclear factors to DNA or induce the recruitment of such factors involved in transcription or chromatin assembly pathways [18].

Histone-DNA interactions are formed primarily by rigid hydrogen bonds between the histone main chain amide and the phosphate oxygen of the DNA. These are strengthened by electrostatic interactions between basic side chains and negatively charged phosphate groups and other nonpolar interactions [19]. While this allows, in theory, nucleosome formation on any DNA sequence, there may be specific sequence preferences for nucleosomal positioning [20]. The nature of the underlying DNA sequences, by which the histone core is wrapped, could be the major determinant of the core histone displacement and the dynamic behavior of the nucleosome under the influence of the SWI/SNF ATPase and sequence-specific transcription factors [21]. The best characterized nucleosomal assembly is the 30 nm fiber, which is stabilized by linker histones [22-24] and the relative positioning of each nucleosome [25], ensuring intimate physical proximity while producing minimal internucleosomal attraction energy [26,27]. Thus, this structure allows dramatic changes in the degree of compaction to occur without a concomitant change in topology. Chromatin is manifest in a number of additionally heightened states of compaction [28], and higher order structures occur upon interaction with non-histone, architectural proteins [29].

In the past three decades, a number of chromatin-related events including DNA methylation, incorporation of histone variants, post-translational modifications of histones, and ATP-dependent chromatin remodeling have been intensely studied. These modifications and the protein complexes involved with their facilitation have been linked to the regulation of many biological processes dependent upon the accessibility of chromatin [30-33]. These include gene expression, DNA repair, chromosome segregation during mitosis, X chromosome inactivation, and chromatin condensation during apoptosis [34-37].

Chromatin modifications impart epigenetic control of gene expression without requisite changes in DNA sequence. Disrupting the balance of epigenetic networks has been linked to severe pathological consequences, including tumorigenesis, syndromes involving chromosomal instability, and neurological disorders [38-40]. Recent advances in our understanding of chromatin structure/regulation and epigenetic inheritance have led to the development of promising new therapies that target the enzymes and complexes that are responsible [41].

## DNA accessibility in transcriptional regulation

The structure of heterochromatin restricts physical access of nuclear factors to the underlying DNA [42]. Regulation of chromatin architecture is, therefore, necessary but not sufficient for controlling gene expression. The activity of sequence-specific activators, repressors, mediator complexes, and general transcription factors are also required to manage transcriptional activity [43,44]. During transcriptional activation, the binding of gene-specific factors to defined DNA sequences triggers a cascade of spatially and temporally coordinated reactions. These result in a chromatin template, appropriately remodeled, which enhances the binding of ubiquitous transcription factors and the general transcription machinery [45,46].

Transcription factors interact with specific sequences and are divided into three classifications. General transcription factors are subunits of the RNA Polymerase II complex, which transcribes template DNA into messenger RNA [47]. The upstream regulatory transcription factors recognize consensus elements located in promoter regions and act by increasing the efficiency of transcription initiation. General transcription factors and upstream transcription factors are ubiquitous factors that require accessible chromatin structure for DNA binding [47]. This is accomplished by the third group of transcription factors which induce the structural remodeling required to open distinct regions of chromatin. These inducible factors are gene-specific and are synthesized or activated at discrete times and in distinct tissues. For example, nuclear receptors, which constitute a large family of ligand-inducible transcription factors, have the capacity to bind to condensed chromatin templates [48]. The response of a given receptor to a particular ligand depends on the set of co-regulators with which it is able to interact. Recruited co-regulators are able to covalently modify histones or remodel nucleosomes in an ATP-dependent manner and these alterations modulate the promoter accessibility to both common transcription factors as well as the basal transcriptional machinery [49]. Ultimately, transcriptional activation results from the integration of specific and ubiquitous factor-binding at the promoter, suggesting that the constitution of the promoter is of critical importance. Thus, the development of tools, such as genome-wide location analysis, will significantly contribute toward a heightened understanding of regulation at this level [50].

## ATP-dependent nucleosome remodeling

Nucleosomal remodeling is an ATP-dependent process that alters chromatin structure in a non-covalent manner [51]. The complexes that facilitate this process are of fundamental importance because they affect the accessibility of DNA to other complexes involved in transcription, DNA repair, and replication. Thus, ATP-dependent chromatin remodeling can affect gene expression, cell cycle progression, and cell differentiation [52].

Chromatin remodeling complexes are divided into several classes, based on the variation within their catalytic ATPase subunit. Although these subunits display homology within the ATPase domain, additional domains vary among classes. For example, the SWI/SNF family contains a bromo domain [53], the ISWI family contains a SANT domain [54], and the Mi-2/NURD family, a chromo domain [55]. Each ATPase associates with different subunits to form distinct multiprotein complexes and each subunit may be differentially involved in the regulation or targeting of remodeling activity.

Nagaich and colleagues studied the interaction between the glucocorticoid receptor and an array of highly positioned nucleosomes, assembled on the mouse mammary tumor virus long terminal repeat. They observed that receptor binding to nucleosomal DNA is enhanced by SWI/SNF and is accompanied by sequential reorganization of histone proteins within the nucleosomes. The action of SWI/SNF is proposed to lead to changes in the position of histone H2B within the nucleosome in concert with the recruitment of GR to a new binding site within the nucleosomal DNA [56]. Recent advances have allowed nucleosome dynamics on promoters to be studied in real time and support the idea that individual nucleosomes may have an inherent capacity to "breathe" [57].

## DNA methylation

Methylation of DNA is a covalent modification that can occur at cytosines within CpG-rich regions of DNA and is catalyzed by DNA methyltransferases [58]. The methylation of DNA affects the binding of proteins to their cognate DNA sequences [59]. Such addition of methyl groups can prevent the binding of basal transcriptional machinery and ubiquitous transcription factors [60]. Thus, DNA methylation contributes to epigenetic inheritance, allele-specific expression, inactivation of the X chromosome, genomic stability and embryonic development [61]. It is through these pathways that progressive DNA methylation is thought to be an agent both of normal aging as well as neoplasias [62].

The majority of methylated CpG islands are located within repetitive elements including centromeric repeats, satellite sequences and gene repeats. These CpG regions are often found at the 5' end of genes where DNA methylation affects transcription by recruiting methyl-CpG binding domain (MBD) proteins that function as adaptors between methylated DNA and chromatin-modifying enzymes [63]. There is a clear relationship between DNA methylation and other silencing mechanisms including histone modifications and chromatin remodeling [64,65]. In fact, several studies suggest that DNA methylation affects genes that are already suppressed by other mechanisms [62].

## Histone modifications

Histone tail alterations encompass the greatest range of variation in epigenetic regulation, encompassing more than 50 known sites of modification [5]. Histones are subject to several forms of post-translational modification, including methylation, citrullination, acetylation, phosphorylation, SUMOylation and ADP-ribosylation [16]. These modifications impart biological consequences by acting as marks for the specific recruitment of regulatory complexes and affecting the structure of the nucleosome. Acting in concert, the combination of different histone modifications is thought to constitute a "histone code" that is interpreted in the form of specific nuclear events [4,66].

Although the interplay among various histone modifications is still largely nebulous, a paradigm is rapidly emerging whereby methylation, acetylation, or phosphorylation at independent sites may work in tandem with other such modifications to convey unique biological consequences [67]. Such crosstalk has already been clearly demonstrated by a number of findings including the cooperation between acetylation and phosphorylation of histone H3 during the cell cycle [68], the correlation between acetylation and argenine methylation in the regulation of estrogen-responsive genes [69], and the competition between methylation and acetylation of histone H3, lysine 9 toward the establishment or disruption of heterochromatin [70]. As new studies continue to highlight the importance of crosstalk in epigenetic regulation, our early understanding of singular histone modifications have yielded to a more delicate model in which minor variations in broad patterns of modifications impart distinct outcomes.

In 1964, Allfrey and colleagues noted a correlation between increased histone acetylation and augmented transcription [71]. Since then, much has been uncovered regarding the affects of histone acetylation and this modification has been implicated in DNA replication, DNA repair, and modulation of chromatin structure [72]. Hyper-acetylation of histone tails at lysine residues is thought to influence transcriptional activity by neutralizing the positive charge of the histone tails and decreasing their affinity for negatively charged DNA, thereby allowing access for transcription factors to promoters in the chromatin [73-75]. Conversely, histone deacetylation is believed to hinder the accessibility of DNA by restoring the net positive charge [76]. In addition to charge-neutralization, more recent studies indicate that histone acetylation/deacetylation regulate transcription by altering higher-order folding properties of the chromatin fiber and providing specific binding surfaces for the recruitment of transcription co-regulators [74].

Near promoter sites, acetylation of histone amino-termini provides binding surfaces for transcription factors of the TFIID transcription initiation complex as well as for proteins in chromatin-remodeling complexes [77]. Agalioti and colleagues have shown progressive acetylation of the human interferon (IFN)-β gene upon transcriptional activation. Each acetylation pattern correlated with the recruitment of a specific protein. The general transcription factors GCN5 and TAF_II_250, the largest subunit of the TFIID complex, are recruited to target promoter regions and sequentially acetylate H4 lysine 8 and H3 lysine 9 and 14, respectively. In turn, H4 lysine 8 acetylation provides a binding site for BRG1 that is part of the SWI/SNF complex that promotes ATP-dependent nucleosome remodeling [77]. In addition to affecting chromatin dynamics through alteration of histone tails, recent studies indicate that acetylation of lysines at the edge of the histone globular domain is also possible and this modification facilitates the recruitment of chromatin remodeling complexes in yeast [78].

The first cloned histone acetyltransferase (HAT) was obtained from *Tetrahymena thermophilia *[79], and sequence similarity with previously identified transcription factors such as CBP/p300, TAF_II_250, and SRC-1, revealed that these transcriptional co-activators all possessed HAT activity [72,80]. These findings strengthen the idea that local acetylation of histones by transcription factors contributes to the activation of promoter-specific gene expression. Histone acetylases act as members of large complexes, such that associating subunits can modulate HAT activity and substrate specificity. In addition, HAT activity can be affected by sequence-specific transcription factors as well as other histone modifications [81]. Homozygous deletions of distinct histone acetylases, *in vivo*, are manifest by disparate developmental defects, suggesting a highly specialized functionality for these enzymes [82].

Antagonism of HAT activity is achieved by a group of enzymes called histone deacetylases (HDACs). Traditionally, these are thought to impart transcriptional repression by catalyzing the removal of the acetyl moiety from histone lysines [81]. The first mammalian HDAC identified is related to the yeast transcriptional regulator, Rpd3 [83]. Since then, additional HDACs have been discovered and appropriately parceled into subclasses, based on sequence homology with their yeast homologs. The human class I histone deacetylases, similar to Rpd3, include HDACs 1, 2, 3 and 8. A second class, including HDACs 4, 5, 6, 7, 9 and 10, are similar to the yeast Hda1 and are regulated through subcellular localization. Class III HDACs, also referred to as the sirtuins, exhibit significant sequence and functional divergence from the class I and II groups [76]. This third class of HDACs displays NAD-dependent deacetylase activity, similar to the yeast Sir 2 protein, and play an essential role in epigenetic silencing [84]. Uniquely, class III HDACs are not sensitive to traditional HDAC inhibitors such as trichostatin A or valproic acid. Although the substrate specificity of distinct HDACs remains nebulous, phylogenetic analysis reveals that HDACs evolved in the absence of histone proteins, suggesting that key HDAC substrates may not be histones [81]. In addition to its classic role, invoking transcriptional repression, contemporary studies have revealed that deacetylation is also required at the promoters of many transcriptionally active genes [85]. Thus, histone deacetylation is an excellent example of the increasingly paradoxical complexities of the "histone code."

Although acetylation of histone tails is largely ephemeral in nature, histone methylation is widely observed to be a mark that confers long-standing epigenetic memory [86]. Mounting evidence suggests that histone lysine methylation is a critical factor in such pathways as transcriptional regulation, X chromosome inactivation, DNA methylation, and the formation of heterochromatin [34-36]. Catalyzed by histone methyltransferases, this modification ultimately mediates either gene activation or silencing, in a residue-dependent manner [86]. The level of specificity is heightened by the variation in biological consequences associated with whether a residue is mono-, di-, or tri-methylated [87,88]. It has also been reported that many transient histone modifications work in tandem with histone lysine methylation, further increasing the potential complexity of this epigenetic modification [11].

Most histone lysine methyltransferases catalyze methyl transfer by way of the SET domain, a module encoded within many proteins that regulate diverse processes, including some critical for development and proper progression of the cell cycle [4,36,89]. Residue-specific histone lysine methylation typically correlates with distinct states of gene expression [90]. Most of the known targeted lysines of histone methyltransferases occur on histone H3 which thereby serves as a conduit of such epigenetic regulation. In general, lysine methylation at histone H3, lysine 9 (H3K9), H3K27, and H4K20 corresponds with gene silencing, whereas methylation of H3K4, H3K36, or H3K79 is associated with actively transcribed genes [90]. Recent evidence implicates histone methylation in the recruitment of chromatin remodeling complexes, as is the case with CHD1, an ATP-dependent chromatin remodeling factor that specifically binds methylated H3K4 [91]. Although once thought to be a permanent modification, enzymes have now been identified that are capable of reversing histone methylation at specific sites [86,92].

The incorporation of histone variants provides yet another echelon to the capacity of epigenetic mechanisms to store of cellular information [37]. Locally, it affects nucleosome structure as well as the propensity of variant-containing chromatin to be remodeled. Hence, histone variant incorporation can alter nucleosome stability, mobility, and potential patterns of histone modifications, likely affecting higher order structure and downstream events [93-95]. For example, a specialized H3-like variant CENP-A, replaces H3 in centromeric nucleosomes to maintain a unique structure that is critical for proper chromosomal segregation [96]. There are many additional studies emphasizing the physiological relevance of histone variants and their significant role in epigenetic regulation [37].

## Non-coding RNA

Accumulating evidence suggests the existence of RNA regulatory networks that are involved in the regulation of gene expression at various levels [97]. It has been observed that non-coding RNA, targeting CpG islands in promoter regions, is able to act in concert with both DNA and histone methylation to affect gene transcription [98-100]. In fission yeast and in *Drosophila*, the involvement of small interfering RNA has been studied in sequence-specific targeting of transgenes, transposable elements, heterochromatin, and some cases of polycomb-mediated gene silencing [101]. Although the current understanding of the influence of non-coding RNA on transcriptional activity is still incomplete, this is an exciting new front in the field of epigenetic modifications that promises to possess answers to broader questions on transcriptional regulation [102].

## Epigenetic aberrations and Cancer

Clearly, the regulation of chromatin structure is a complex and dynamic process. It is modulated at several levels by distinct mechanisms such as DNA methylation, nucleosome remodeling, histone post-translational modifications, incorporation of histone variants, and non-coding RNA. Aberrations in such epigenetic mechanisms are likely to impact gene expression as well as other physiologically critical processes such as chromosome condensation, segregation, and apoptosis.

Several lines of evidence indicate that tumorigenesis in humans is a multistep process in which a succession of genetic changes leads to the progressive conversion of normal cells. While genetic alterations can account for some of theses changes, many of the alterations in gene expression observed with cancer are caused by epigenetic modifications [103]. These observations highlight the relevance of epigenetic mechanisms toward the establishment of proper cellular function. Misregulation of these mechanisms cooperates with genetic mutations and contributes to the establishment and progression of neoplastic diseases.

Although a loss-of-function for a remodeling complex subunit is not likely sufficient to induce oncogenesis, such an abnormality could enhance the cascade of events leading to oncogenic transformation, when exhibited in tandem with specific genetic mutations [104]. Alterations of remodeling complex activity in various mammalian cells and organs was correlated to differential global and site-specific genomic methylation patterns [105-107] as well as to impaired histone post-translational modifications [108]. These observations underscore the importance of chromatin remodeling factors in the regulation of gene expression during development and in disease [109]. In one example, Brg1 null mice lack the functional ATPase catalytic unit of the SWI/SNF remodeling complex and are embryonic lethal [110]. In adults, altered expression of Brg1 is observed in subsets of lung, breast, prostate, and pancreatic cancers. Additionally, in the familial cancers termed the "rhabdoid predisposition syndrome," predisposition is inherited through specific inactivating mutations of the SNF5 subunit present in all SWI/SNF complexes [111]. Other such mutations to chromatin remodeling complexes have been associated with oncogenesis and much effort is being allocated toward the potential for therapeutic intervention at this level [112].

Imbalance of histone acetylation/deacetylation in promoter regions contributes to the deregulation of gene expression and has been associated with carcinogenesis and cancer progression [113,114]. Both, histone acetylases and deacetylases have central roles in regulating the access and recruitment of transcription factors to DNA regulatory elements and in the regulation of other post-translational modifications at the lysine residues. The high conservation of acetylase/deacetylase complexes illustrates the importance of their function in cell proliferation and differentiation. Translocation, amplification, over-expression, or mutations of HAT genes occurs in a variety of human pathologies [80,115,116] and chromosomal translocations that lead to the fusion of transcription factors to HATs or HDACs have been linked to hematological malignancies such as certain leukemias [115].

The aberrant targeting of HAT or HDAC activity to specific gene promoters can result from the fusion of transcription factors with protein domains that retain co-repressor or co-activator binding capacity. Acute promyelocytic leukemia and acute myeloid leukemia are caused by chromosomal translocations leading to the expression of transcription factors fused to the nuclear receptor RAR or to the zinc finger nuclear protein ETO, respectively, which contain co-repressor interaction domains [117,118]. The progression of these leukemias is linked to the abnormal recruitment of the N-CoR/SMRT co-repressor complex containing histone deacetylase activity which acts by blocking differentiation and allowing uncontrolled growth of hematopoietic cells [117,118]. More recent studies demonstrate that the transcriptional repression of target genes by fusion proteins in leukemia is reinforced by epigenetic modifications such as DNA methylation. These epigenetic marks are then maintained throughout multiple cell divisions [119].

The misregulation of DNA methylation is another epigenetic irregularity known to contribute to the initiation and progression of tumorigenesis [120]. Indeed, changes in the pattern of DNA methylation were correlated with altered histone post-translational modifications and genetic lesions. Either hypermethylation or hypomethylation have been identified in all types of cancer cells examined, to date. Hypomethylation at centromeric repeat sequences has been linked to genomic instability [121] whereas local hypermethylation of individual genes has been associated with aberrant gene silencing [122]. In oncogenic cells, hypermethylation is often correlated with the repression of tumor suppressor genes while hypomethylation is associated with the activation of genes required for invasion and metastasis [123-127]. New techniques, such as the polymerase chain reaction amplification of bisulfite-modified DNA, have enabled the study of patterns of DNA methylation. This method is currently being improved and adapted for cancer cell identification, profiling of tumor-suppressor-gene expression, and prognostic factors that are linked to CpG island hypermethylation [128-130]. The DNA methylation patterns may become invaluable in cancer patient prognosis and its potential as a biomarker is currently under investigation [131].

Accumulating evidence implicates the aberrant loss or gain of histone methyltransferase (HMTase) activity in tumorigenesis. For example, mice which fail to express the H3K9-specific HMTase, SUV39H1, are subject to heightened chromosomal instability and consequent oncogenic potential [132]. Conversely, it is over-expression of Smyd3, an H3K4-targeting HMTase, that has been linked to proliferation of tumor cells [133]. Since the initial finding, linking Smyd3 to hepatomas and colorectal carcinomas, a polymorphism involving a transcription factor binding element in the upstream regulatory sequence for Smyd3 has been linked to a heightened risk for oncogenesis [134,135]. Consequently, suppression of Smyd3 expression has been the subject of several recent therapeutic studies [136-138].

Apart from their ability to covalently modify histones, two histone methyltransferases have been shown to methylate the p53 tumor suppressor, directly. Set9, which methylates H3K4 [139,140], has also been implicated in the regulation of p53 by methylating that protein at lysine 372. Methylation of this site stabilizes p53 and limits its localization to the nucleus [141]. More recently, Smyd2, which methylates H3K36 and augments proliferation of NIH3T3 cells [3], has also been directly linked to the regulation of p53. By methylating lysine 370 of p53, Smyd2 inhibits the activity of that protein in transcriptional regulation [142]. Current and future studies on the ability of HMTases to act directly on oncoproteins and tumor suppressors will undoubtedly open an exciting new frontier in therapeutic intervention.

Assuming that epigenetic changes do not solely affect protein expression but also the expression of non-coding RNAs, anomalous epigenetic regulation may have drastic impacts on biological processes involving regulatory RNAs [143]. Analyzing non-coding RNA profiles revealed that distinct patterns were associated with specific cancer types, developmental lineages, and differentiation states of the tumors [144]. A range of evidence supports that micro-RNA profiling will be useful in diagnosis, prognosis, and management of human cancers in the near future [145-147]. However, the precise role of non-coding RNA in the generation, maintenance, and progression of tumors remains to be determined as does the link between variations in non-coding RNA profiles and epigenetic alterations.

## Conclusion

Although chromatin states, once initiated, can be epigenetically maintained and inherited, several studies support that epigenetic control of gene expression may be altered by environmental stressors/toxicants or carcinogens. These alterations may, in turn, compromise genome integrity and stability. Clearly distinguished from genetic mutations, these epigenetic alterations have been termed "epimutations" and must be actively maintained, in contrast to genetic mutations, which are inherited passively through DNA replication [148]. Such epimutations rarely appear in healthy tissues, indicating that epigenetic therapies may have high tumor specificity. Furthermore, in contrast to genetic deletions, causing irreversible loss of gene function, epigenetic modifications are reversible, making them attractive targets for therapeutic intervention [149]. To restore normal expression of tumor suppressors, by reversing these epimutations, has consequently become a new therapeutic ambition in cancer treatment. Indeed, aberrant gene silencing mediated by DNA methylation and histone deacetylation can be reversed by DNA methyltransferase inhibitors [150] and histone deacetylase inhibitors [151], respectively. In many tumor cell lines, promising results have been obtained after treating cells with such pharmacological agents [152-155]. Resetting normal patterns of gene expression is often achieved and cell differentiation or apoptosis is restored. However, preliminary results of ongoing clinical trials suggest that the outcome of such treatments depends on the exact defects of the cancer cell itself, which can be a combination of genetic and epigenetic changes, such that tandem implementation with other anticancer therapies may be most successful.

In the last decade, great strides have been made toward our understanding of chromatin structure and its role in the regulation of nuclear processes. Recognizing patterns of histone post-translational modifications, deciphering the relationship between these modifications and DNA methylation, and characterizing the relevance of epigenetic alterations in neoplasias encompass a new frontier in the etiology of cancer. Thus, the examination of epigenetic aberrations is sure to be a progressively critical factor in the diagnosis and treatment of malignancies.

## Competing interests

The author(s) declare that they have no competing interests.

## Authors' contributions

M.D. and M.B. wrote and finalized the manuscript. All authors read and approved the final manuscript.
